# The Mechanisms of Mating in Pathogenic Fungi—A Plastic Trait

**DOI:** 10.3390/genes10100831

**Published:** 2019-10-21

**Authors:** Jane Usher

**Affiliations:** Medical Research Council Centre for Medical Mycology, Department of Biosciences, University of Exeter, Geoffrey Pope Building, Exeter EX4 4QD, UK; j.usher@exeter.ac.uk

**Keywords:** mating, meiosis, genomes, MAT (mating type) locus, *Candida*, Aspergillus, Cryptococcus

## Abstract

The impact of fungi on human and plant health is an ever-increasing issue. Recent studies have estimated that human fungal infections result in an excess of one million deaths per year and plant fungal infections resulting in the loss of crop yields worth approximately 200 million per annum. Sexual reproduction in these economically important fungi has evolved in response to the environmental stresses encountered by the pathogens as a method to target DNA damage. Meiosis is integral to this process, through increasing diversity through recombination. Mating and meiosis have been extensively studied in the model yeast *Saccharomyces cerevisiae*, highlighting that these mechanisms have diverged even between apparently closely related species. To further examine this, this review will inspect these mechanisms in emerging important fungal pathogens, such as *Candida, Aspergillus*, and *Cryptococcus*. It shows that both sexual and asexual reproduction in these fungi demonstrate a high degree of plasticity.

## 1. Introduction

There are approximately 1.5 million identified fungal species, of which a few hundred have been reported or suspected of being the causative agent of disease in humans [1]. Human fungal pathogens can cause a myriad of disease, life-threatening infections, chronic infections, and recurrent superficial infections [2]. On a global scale, the incidence of fungal infections is rising, affecting millions of people per annum. The most prevalent species known to be causative agents are *Candida, Aspergillus*, and *Cryptococcus,* with *Candida* being the most prevalent [3]. These fungi are part of the most populated phyla of the fungal kingdom, the Ascomycota, with *Cryptococcus* belonging to the Basidiomycota, in which numerous pathogens of both animals and plants are present [4]. It is clear that microbial pathogens have emerged not only independently in different phyla of the kingdom but also multiple times independently within the phyla [4]. 

Sexual reproduction is a common attribute to many fungi; a general evolving theme in fungal genomics is that there appears to be very few, if any truly asexual fungi [5]. Each genome sequenced and annotated to date has revealed that the machinery for both mating and meiosis are conserved (Table 1) [5,6,7,8,9,10,11,12,13,14]. Therefore, it can be concluded that for pathogenic fungi at least, a sexual stage is present and, in many cases, remains to be discovered under laboratory conditions. The rarity or cryptic nature of these sexual cycles leads to clonal populations with limited recombination events [15], thus having broad implications for the evolution of eukaryotic microbial pathogens. It should also be noted that exclusively clonal fungi are scarce, with molecular markers often revealing the occurrence of at least some degree of recombination having occurred [11,16,17,18]. Apart from footprints of recombination based on population genetics data, a further type of evidence indicative of a sexual cycle having occurred in most fungi comes from the apparent functionality of the mating type genes [6,8,19], even seen in species without known sexual structures.

Studies in the model yeast *S. cerevisiae* have played a central role in elucidating the regulation machinery involved and the significance of sexual cycles [20,21,22,23,24,25,26,27,28]. Yet, despite the wealth of information from these systems, it is now recognised that it is a surprisingly plastic trait in different species [8]. In general, most ascoymcetes exhibit mixed reproduction systems, with indications of sexual and asexual reproduction. However, a major problem distinguishing these events is the difference between haploid selfing and clonality in homothallic fungi using common population genetic approaches. 

## 2. Meiosis in the Model Yeast *S. cerevisiae*

**Figure 1:**
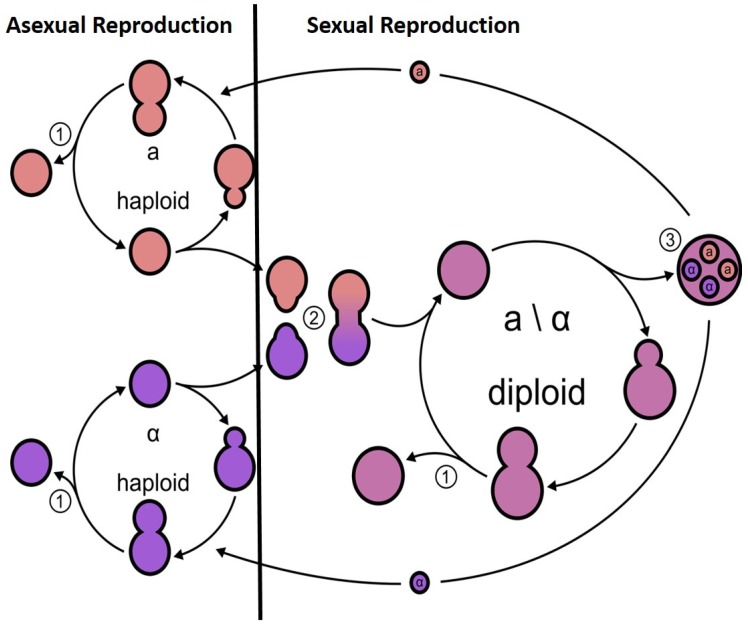
Schematic of the life cycle of *S. cerevisiae*. *S. cerevisiae* cells of opposite mating types (a or α) are found as haploids and have an asexual reproduction via budding (1), producing a daughter cell. Haploid cells in a mixed population can secret pheromone to attract cells of the opposite mating type, resulting in ‘schmooing’ of cells followed by conjugation (2). Under stress conditions, the diploid cells will undergo sporulation (3) and meiosis resulting in four competent haploid recombined progenies.

Mating in fungi was initially and best studied in *Saccharomyces cerevisiae*; however, this is not a true representation for other ascomycetes. Briefly, mating in *S. cerevisiae* occurs between two haploid cells of different mating types, **a** and α cells, producing diploid a/alpha cells [28]. *S. cerevisiae* cells can be found to exist as three main mating types: **a,** α, and the diploid state **a/**α. Under favourable conditions, mating occurs between haploid **a** and α cell types, generating an **a/**α diploid (Figure 1). Mating is stimulated by the presence of a pheromone, which binds to the Ste2 receptor in **a** cells or the Ste3 receptor in α cells. For example, **a** cells’ mating pheromone ‘a-factor’ signals the **a** cell presence to neighbouring α cells, and the cells respond by growing a ‘shmoo’; the classical growth projection with the distinctive peanut shape, as a result of cells responding to the mating factor, towards the source of the a-factor pheromone. The response of haploid cells only to the mating pheromones of opposite mating types facilitates mating between **a** and α cells but in general not between cells of the same mating type. The phenotypic difference between **a** and α cells is due to specific sets of genes being transcribed and repressed in the different mating types. These different sets of genes that characterise **a** and α cells are due to the presence of one of two alleles *HML* or *HMR* and the MAT locus on chromosome III and the HO endonuclease on chromosome IV. The genetic regions at *MAT**a*** and *MATα* have been characterised, showing that the two regions are not the same length and they encode regulatory proteins that control **a**-specific or α-specific genes [29]. The two regions show little homology and are therefore not true alleles. The proteins encoded by the *MAT* locus are DNA-binding proteins, which define one of three possible cell types: Haploid *MAT**a**, MATα*, or diploid [30,31]. *MATα* encodes two genes α1 and α2 and determines whether a cells exhibit an a or α phenotype. *MATα1* controls the expression of α-specific genes and *MATα2* suppresses the expression of a-specific genes that would be expressed constitutively [31]. The *MAT**a*** locus encodes a single protein a1, which is dispensable for mating but acts in conjunction with *α2* to control gene expression after successful mating in the diploid cell state [29]. The production of ‘shmoos’ and in fact mating in *S. cerevisiae* occurs via an all-or-none, switch-like mechanism, therefore preventing the cells from making unwise and energy-inefficient mating. *MAT**a*** haploids express the genes a1 and a2 from the *MAT* locus while *MAT*α haploids express α1 and α2 from *MAT*. Diploid cells are heterozygous at the *MAT* locus, are able to sporulate, and do not respond to pheromones. The diploid cells are unable to mate but undergo meiosis in response to environmental cues to sporulate and undergo a reductive division to result in four haploid cells (Figure 1). The majority of yeast laboratory strains are heterothallic with stable mating types. However, some strains carry an active *HO* gene and are homothallic, indicating that, as haploid cells, they are able to switch mating type via changes of the genetic composition of the *MAT* locus to that of the opposite mating type. The descendants of the original cell can therefore mate and will form non-mating diploids with a silenced *HO* gene. In addition to the *MAT l*ocus, many strains also carry two complete but unexpressed copies of the mating-type genes at the silent loci *HML* and *HMR* carrying α information and a-specific sequences, respectively. The mechanism of silencing *HML* and *HMR* loci is mediated by Sir2, a histone deacetylase, and its associated proteins. The mating-type switching process is stimulated by the *HO* endonuclease. 

A key regulator that mediates this cell-type specificity is the a1/α2 heterodimer that represses expression of the genes involved in mating (Table 1). a1/α2 also inhibits the expression of RME1, a protein that encodes a negative regulator of meiosis [32]. RME1 expression is crucial in preventing **a** or α cells from initiating meiosis. IME1 is the direct target of RME1, encoding a transcription factor that in conjunction with UME6, regulates the downstream meiotic genes [33]. IME1, is a master regulator of meiosis, with multiple pathways converging, such as cell-type and nutritional signals [27,33,34,35,36]. Of notable importance is IME4, whose expression is induced under starvation conditions and can activate IME1 through modification of its mRNA [37]. Downstream of IME1 is IME2, a serine/threonine kinase that positively regulates subsequent events in meiosis. IME2 also negatively regulates IME1 by targeting it for degradation [38], therefore restricting IME1 to a narrow window of activity. 

The first division in meiosis is reductive in nature, with the homologous chromosomes segregating from each other: Meiosis I. The second meiotic division is equational with the separation of the sister chromatids: Meiosis II. A conserved complex of cohesions holds the sister chromatids together during meiosis I, REC8 provides meiosis-specific cohesion activity, and the cleavage of this protein is essential to allow the separation of the sister chromatids during meiosis II [39]. Meiotic recombination plays an active role in the pairing of homologous chromosomes. Central to meiotic recombination is the formation and repair of DNA strand breaks introduced by SPO11, a highly conserved protein (Table 1) [25]. In addition, accessory factors can promote DSBs formation and strand exchange, although these factors are poorly conserved in other fungal species [40].

## 3. Benefit of Sexual vs. Asexual Reproduction

An emerging theme is that programmes of sexual reproduction exhibit a remarkable degree of plasticity [11]. This is true of meiosis, where recent studies have revealed marked differences between non-pathogenic and pathogenic species, and even between those considered to be closely related [5,13,14,41,42,43]. Such studies highlight that the differences between species are likely to be as important as the similarities. They also hold a word of warning that inferring information from related species should always be approached with a level of caution, as not all genes can functionally complement each other [19,44,45,46]. Despite the prominence of sexual cycles in many eukaryotic species, the benefits of such a strategy are continuously debated. As many of the fungi are saprophytic and require no interaction with a host, the mating system is not driven by a need to generate variability in the population to evolve; however, the yeast requires that ability to respond to changes in its environment and a sexual cycle may provide the means to do so. One current understanding is that the ability of sexual reproduction is to promote genetic variation important for the survival of lineages [47]. This promotes adaptation to varied environments and can also limit the accumulation of deleterious mutations [9,48]. That said, sexual reproduction also has associated increased ‘costs’ for the cell; asexual reproduction is predicted to be advantageous under certain conditions as it can have short-term evolutionary advantages. Sexual reproduction also carries the risk of genetic conflicts and a potential loss in well-adapted genetic combinations, for example, multidrug resistance [49,50,51]. 

As a point of generalisation, the main function of a sexual cycle is to repair DNA and the production of higher quality progeny [52]. In fungi, meiosis allows for DSB repair in the form of cross-over events, only with diploid cells, with the products of two mated haploids being capable of this process. 

The benefit of sexual vs asexual reproduction is further complicated in pathogenic fungal species. The issue of host–pathogen interactions also ought to be considered, which can lead to co-adaptation and species that have an increased rate of genetic variation. Such interactions have two potential outcomes: (i) Sexual reproduction in the host promotes adaptation, whereby the host can escape by co-evolving with the pathogen; and (ii) in asexual reproduction, the host is unable to outrun the pathogen, leading to disease [52]. Amongst the eukaryotes, sexual reproduction is ubiquitous and asexual reproduction is often viewed as an evolutionary dead end due to the potential accumulation of deleterious mutations [52]. In relation to human fungal pathogens, *Candida albicans* and *Aspergillus fumigatus*, for many years they were considered to be obligate asexual species. However, analysis of their genomes has revealed that they have retained many genes associated with mating and meiosis (Table 1). They contain a mating type (*MAT* locus), a locus encoding the transcription factor that is the master regulator of sexual reproduction [12,25,43,53]. In *C. albicans, C. neoformans*, and *A. fumigatus*, the existence of sexual or parasexual cycles has now been clearly established [12,14,25,43,53]. What is unique about each species is that they mostly reproduce clonally, with limited conditions available for sexual reproduction. This poses the question: Why is it that certain species have retained the machinery and genes for a sexual cycle given the fitness costs to the cells? 

## 4. Mating Type-Loci and Mate Recognition

**Figure 2:**
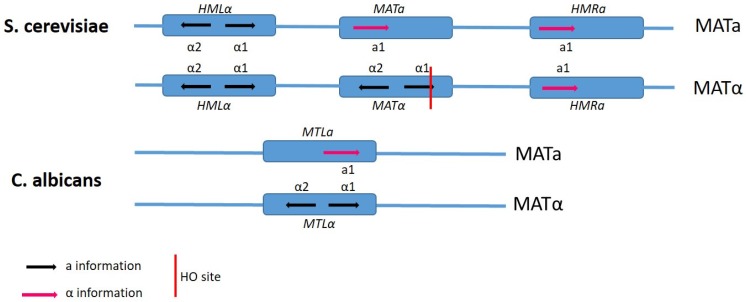
A schematic representation of the mating-type loci in *S. cerevisiae* and *C. albicans. S. cerevisiae* has three mating-type loci; *HMLα, MAT**a*** or MATα, and HMRα compared to one *MLT* in *C. albicans*. The *MAT* and *MTL* regions determine the mating type. In *S. cerevisiae*, the mating-type loci are all found on chromosome 3 and chromosome 5 in *C. albicans*. There is also no *HO* endonuclease site in *C. albicans.*

Generally, the expression of pheromone and pheromone-receptor genes is regulated by the MAT-encoded transcription factors [54,55]. In *C. albicans* the mating type-lie (*MTL*) locus has undergone expansion since its diversion from the related model yeast *S. cerevisiae* [25,53,56,57,58,59]. In *C. albicans*, the *MTL* locus includes three genes (Figure 2) that are not present in the *S. cerevisiae MAT* locus, two of which are essential for growth and play roles in cellular processes not related to reproduction. In *C. albicans*, ***a**1* and *α2* transcription factors are encoded at the *MTL* locus and control sexual mating by inhibiting a unique phenotypic switch that is required in conjugation.

## 5. Pathogenic Fungi

Pathogenic fungi must evolve to evade the immune response of the host in addition to maintaining its own structural and genetic integrity. Due to the variety of different fungal pathogens that affect humans and plants, it is likely that the ability to be a causative agent of disease arose multiple times within the lineage. As previously stated, not all of these organisms have a sexual cycle even those that have retained the genetic components remain elusive in the detail of their meiotic cycles.

## 6. *Candida albicans*

**Figure 3:**
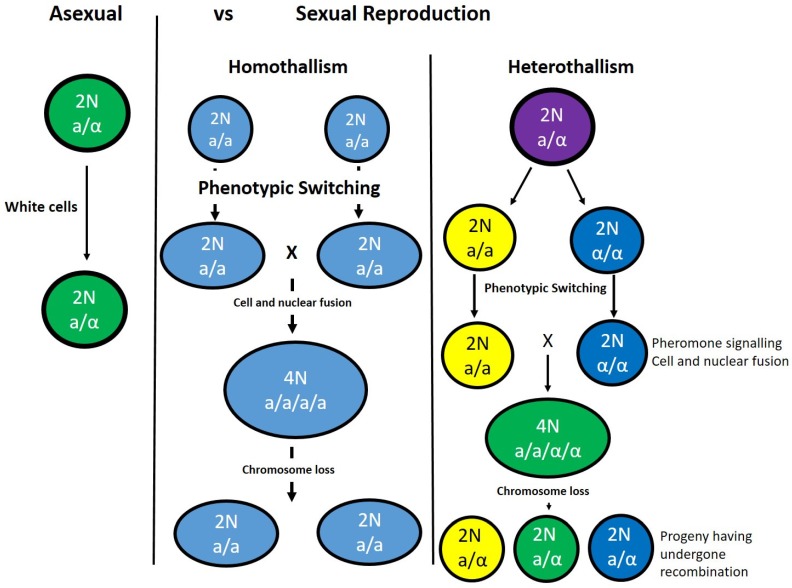
*Candida albicans* reproduction. *C. albicans* is generally diploid (2N) in nature and can reproduce either asexually or sexually—either homothallic or heterothallic mating. To become mating competent, MTLa and MTLα cells must switch from white to opaque. The opaque cells secrete pheromone that will result in the formation of conjugation tubes, allowing for cell and nuclear fusion and a tetraploid cell (4N). Homothallic mating is driven by the loss of Bar1 in MTLa cells. The tetraploid cells (4N) are unstable and undergo chromosome loss to return to a diploid state (2N); in heterothallic mating, this will result in recombinant cells with the potential to have increased levels of drug resistance and fitness advantages.

Perhaps the most prevalent and well-studied fungal pathogen of humans, *Candida albicans*, is an example of a fungus where the host is essential [60]. It has long been believed that *C. albicans* was asexual and no sexual cycle at all [53]. It was not until the genome was sequenced that evidence of the genetic components for sexual reproduction were identified and investigated further [53]. It was determined that the *C. albicans* genome contained many of the regions similar to those in *S. cerevisiae*, as discussed above (Table 1). In *C. albicans*, these regions are named *MTLa* and *MTLα*, encoding regulatory regions similar to those in *S. cerevisiae*, with the exception of over a larger region in the genome (Figure 2) [58]. Within these regions, many of the key players in a sexual cycle, such as transcriptional regulators, pheromone receptor genes, and loci organisation, were found [58]. It has since been shown that the pheromone response induces the expression of genes, with homologs in *S. cerevisiae* classified as having roles in mating [58]. Therefore, with a genome possessing the main components for mating and meiosis, the question remained: Does *C. albicans* undergo a sexual cycle? This question does not have a straightforward answer, but rather lies within the relationship between *C. albicans* and its host. In the laboratory, *C. albicans* can grow as either white or opaque colonies independent of the mating type (Figure 3). The switch from white to opaque colonies is regulated by homeodomain proteins at the mating type locus [61], with the rate of switching a relatively rare event. Mating has been observed in clinical isolates and is related to mating efficiency and virulence, whereby the most robust cells are those least likely to mate [53], most likely due to the energy costs associated with sexual reproduction. 

Generally, *C. albicans* exists as a diploid yet the genes involved in mating are only present in a single copy [53], and the commonly used lab strain is heterozygous for the mating alleles. In a study by Miller and Johnson, [61], engineered lab strains were studied in response to each other, and the same nomenclature to that of *S. cerevisiae* was used, with cells deemed **a** and α. The strain produced opaque colonies; however, the diploids were not observed to undergo switching. The cells were observed to secrete pheromone, with the alpha cells producing pheromone that induced projections in the **a** cells towards the α cells. This was only observed in the opaque a-type cells and not in the α cells, indicative of the pheromone response being cell-type specific [58,61]. The opaque cells were also observed to have an increased efficiency at mating compared to the white cells. However, opaque cells are noted to be less stable in the host, thus supporting the hypothesis that *Candida albicans* do not mate within the human host. However, if the human body is approached as a variety of niches rather than a single entity, it has been observed that this no human host mating theory is only true for specific niches of the body and is influenced by the presence of other microbes [62]. For example, white-opaque switching is induced in the gastrointestinal tract, where there is competition for nutrients from bacterial inhabitants and the stressful environment encountered by the cells [63]. Therefore, it is possible that the presence of other microbes and the molecules that they secrete can have an influence on the sexual cycle of *C. albicans*. A third phenotype of *C. albicans* has recently been described [41], a ‘gray’ colony formation. This has not been observed in all clinically isolated, the switch is white-gray-opaque, and is independent of regulation by the mating-type locus, with strains displaying this ‘triphenotype’ intermediate in their mating competency compared to those of the opaque and white phenotypes. It is hypothesized that this third phenotype is the result of establishing niche-specific switching within the human host [41,61,62,63,64,65]. 

The challenge for this human pathogen is to be able to evade the host immune system and compete with a plethora of microbes living within the same host [25]. *C. albicans* must be able to survive within the host and respond to the host’s immune system. Ascospores are antigenic, therefore a sexual cycle within the host could result in a greater and effective immune response [6,25]. Therefore, *C. albicans* has been termed a parasexual cycle, as this allows for DNA repair created by recombination to occur without host recognition (Figure 3). In such a system, tetraploids are highly unstable and lose chromosomes to generate stable diploids [25]. As a result, genetic recombination occurs at a much lower rate than in the meiotic cycle of *S. cerevisiae*, but at a level where sufficient variation is generated within the population. Despite the obvious lack of meiosis, the parasexual cycle in *C. albicans* does help to increase diversity and goes part way to explain the maintenance of the mating-type genes being retained in the genome. 

## 7. Aspergillus Fumigatus

*Aspergillus fumigatus* is a human pathogen of predominantly immunocompromised patients. Due to this clinical importance, it has been studied in depth for many years. It was first described over 150 years ago and is commonly found in soil, with human infection having a high mortality rate [66]. Initially it was thought to only reproduce via mitotic spores, although mating-type loci have been identified in the genome. HMG-domain genes similar to those found in other sexually reproducing species in addition to pheromone and pheromone receptors [43,67] have been mapped. The possibility of sexual reproduction has been further strengthened by the identification of two versions of the mating-type locus: *MAT1-1* and *MAT1-2*. However, the study of isolates from the environment and clinics revealed an almost equal ratio of the two mating types present, which could be seen as a lack of sexual reproduction [12]. 

**Figure 4:**
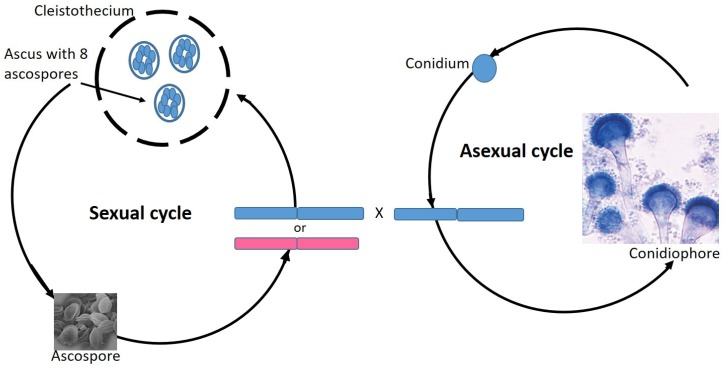
Reproduction in *Aspergillus fumigatus*. Asexual reproduction in *A. fumigatus* is via mitotic division of haploid (1N) cells or through the formation of asexual conidiospores. Sexual reproduction is heterothallic between *MAT1-1* and *MAT1-2* mating types. The mating product are cleistrothecia containing multiple ascospores.

That said, there has been evidence for recombination and gene flow from population genetic studies [12], leading to experiments in the laboratory setting using strains of the opposite mating types. These showed that the fungus is able to mate with a strain of the opposite mating type. This body of work showed that *A. fumigatus* does indeed possess a fully functional sexual reproductive cycle that leads to the production of ascospores. The presence of this sexual cycle in a medically important fungus is an invaluable tool for forward genetic analysis and research into the genetic basis of its pathogenicity and drug resistance. A further study [49] revealed that there is no prevalence for one mating type over the other in clinical isolates, indicating that the mating-type locus plays a yet to be discovered role in strain virulence. These studies highlighted a variety of molecular tools for teasing apart the sexual cycle for organisms that appear to be asexual in nature [12]. A caveat of this study is that to date the ability of *A. fumigatus* to mate within the human host has yet to be observed. Similar to that of *C. albicans*, mating within the host may only be occurring as a result of exposure to specific physiological cues or the inverse may be the case and the fungus, given its success as a pathogen, loses the ability to mate in the host and reproduces only by mitosis (Figure 4). 

## 8. Cryptococcus Neoformans

**Figure 5:**
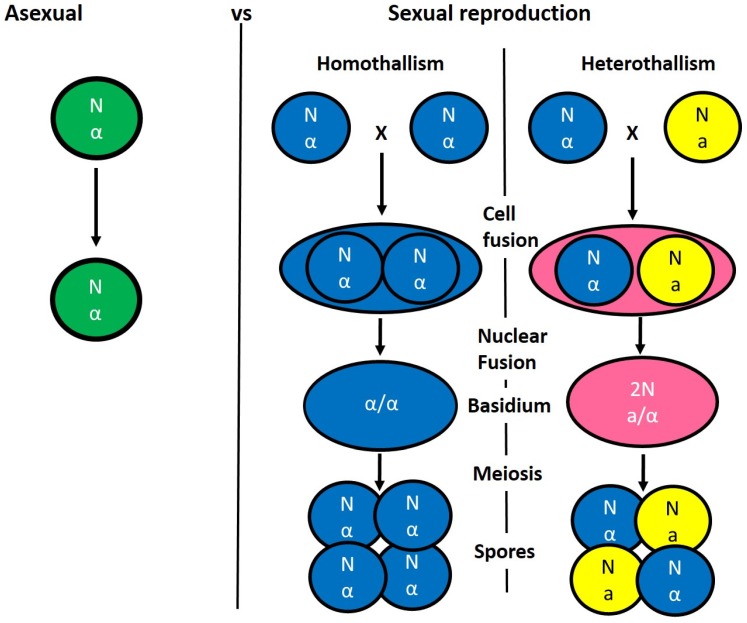
Reproduction in *Cryptococcus neoformans*. *C. neoformans* cells are generally haploid (N) and divide asexually (left panel) or enter a sexual cycle, (right panel). In the heterothallic mating, pheromone secreted between a and α cells results in cell fusion, where the nuclei do not fuse but form a filament called a dikaryon. The cell tips differentiate into basidia (2N). This is the site of nuclear fusion and meiosis. Multiple haploid cells are produced following further rounds of mitotic divisions. A sexual cycle in *C. neoformans* results in hybrid recombinant strains with increased drug resistance and fitness advantages.

*C. neoformans* is an opportunistic human fungal pathogen that can undergo bisexual and unisexual mating; a homothallic sexual cycle that induces a yeast to hyphal dimorphic transition in α mating type cells which will be discussed herein. *C. neoformans* can undergo mating and fruiting, both of which involve meiosis under desiccation or nitrogen starvation conditions [14]. C. neoformans is found primarily in the lungs of patients, in addition to many environmental niches such as, soil, decaying wood and bird droppings, and worldwide, it is the largest source of fungal meningitis [14,67]. In an infectious state, the basidiospore or desiccated cell is small enough to travel deep into the lungs of patients and seed infection. In suitable environmental conditions, not within the host, sexual reproduction has been observed [14,67]. Under favourable mating conditions, fusion occurs between cells of the opposite mating type, resulting in dikaryotic hyphae that are characteristic of the Basidiomycota. The mating type, either **a** or α, is defined by the genetic components of the mating type locus in the bipolar mating system of *C. neoformans* (Figure 5) [68,69]. Numerous pheromone receptor genes have been identified in the genome; unlike other genomes, they are not tightly linked but dispersed throughout the mating type locus [70]. Within the α mating-type allele, a novel protein has been identified, with a complementary protein being identified in the mating-type locus and has been observed to interact with each other to regulate mating genes [71]. 

Unique to *C. neoformans* is the genome arrangement of genes associated with mating. The evolution of the α mating-type locus is similar to that of mammalian sex chromosomes, with large rearrangements and recombination suppressed, resulting in a large region of sex-determining genes that are generally unlinked [14,69,70,71]. From an evolutionary standpoint, this shows a shift towards sex chromosomes rather than mating-type loci. The **a** and α cells have no physiological differences; however, they do behave differently and are found at different proportions in clinical isolates. To date, the majority of clinical isolates are the alpha mating type [70]. While *C. neoformans* is capable of sexual reproduction, independent evolution of the mating loci within the two mating types resulted in one mating type being able to establish infection independently of the other. To such an extent that mating and meiosis between two α cells has been observed [71], this may also influence why the majority of clinical isolates are of this mating type. Within a host, this may also be advantageous by allowing for the benefits of sexual reproduction caused by the host’s response without the need to find a compatible mating partner. The lack of a requirement for a compatible mate has removed the necessity for the metabolic costs associated with sexual reproduction. 

## 9. The *Nakaseomyces* Genus

**Figure 6:**
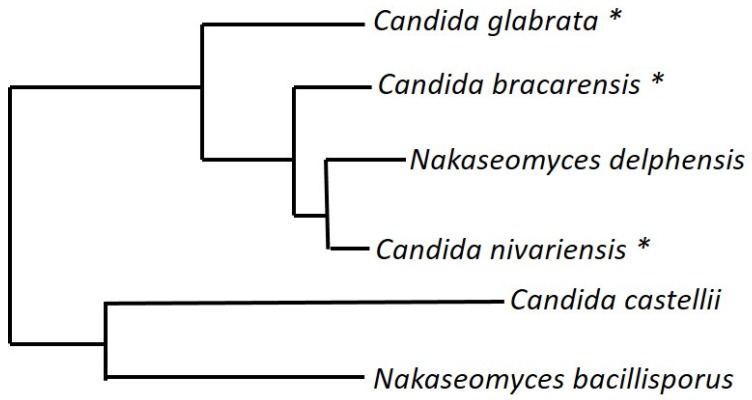
The *Nakaseomyces* genus. The six species that comprise the *Nakaseomyces* genus, three of which marked with * are pathogenic. This clade is separate from the CTG-clade compromising of the other *Candida* species.

*C. glabrata* is phylogentically closer to *S. cerevisiae*, the model yeast, than to other better-known *Candida* species, such as *C. albicans*, and is part of the *Nakaseomyces* genus. This genus is currently made up of three yeasts isolated from the environment: *Nakaseomyces delphensis, Candida castellii*, and *Nakaseomyces bacillisporus*, along with two other pathogens, *Candida nivariensis* and *Candida bracarensis* (Figure 6). The members of this genus are both sexual and asexual in nature, with *C. glabrata* and *N. delphensis* containing the *HO* gene and MAT-like loci, highlighting the plastic nature of reproductive cycles. 

The genomes of all six species have been sequenced and annotated and compared to *S. cerevisiae* [16]. As a general theme, all *Nakaseomyces* nuclear genomes are small, free from transposons, and contain less genes than *S. cerevisiae*. In contrast, their mitochondrial genome, with the exception of that of *C. glabrata*, are large and contain palindromic elements and GC inserts [16]. The number of chromosomes ranges from 8 in *C*. *castellii* to 15 in *N. bacillisporus*; the more classically referred to ‘glabrata group’ has less variation, with between 10 and 13 chromosomes revealed by pulsed field gel electrophoresis [16,72]. In addition to the variable chromosome numbers, comparative analysis of the genomes revealed several gene losses and gain of species-specific genes when compared to *S*. *cerevisiae* [73]. These *Nakaseomyces*-specific events were used in analysis to determine if these events were evolutionarily specific to the *Nakaseomyces* group or specific to the ‘glabrata group’ or indeed to *C. glabrata* itself. In addition, the *Nakaseomyces* clade is supposed to share specific metabolic traits with *S. cerevisiae*, such as the ability to ferment sugars, growth under anaerobic conditions, and the formation of petite mutants. However, in-depth sequence analysis has revealed that all the *Nakaseomyces* lack NADH:ubiquinine oxidoreductase complex and all are gal minus; therefore, they are unable to assimilate the six-carbon sugar galactose, a gene loss that has been confirmed in the laboratory [74]. This genus can be subdivided into two main lineages: The first containing *C. castellii* and *N. bacillisporus*, having followed a divergent evolution; and the second group containing the three pathogenic yeasts and *N. delphensis*, closely related to *C. bracarensis* and *C. nivariensis*. These subdivisions are indicative of the genus having undergone several independent evolutionary events, resulting in the emergence of human pathogens. 

**Figure 7:**
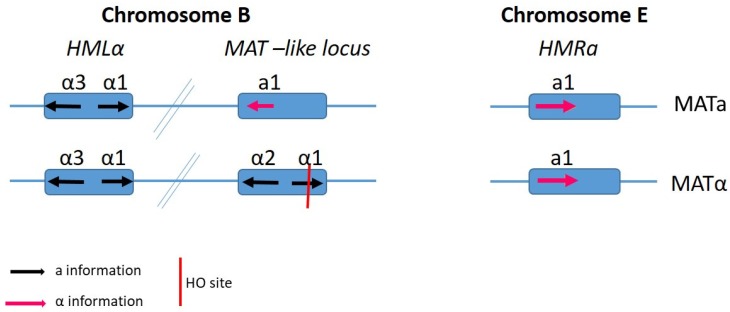
Schematic of the mating-type like (MTL) loci in *Candida glabrata.* There are three mating-type loci similar to that of *S. cerevisiae*. The *MAT*-like loci (*MTL*) determine the strain mating, with the *HMR* and *HML* encoding **a** and α information. The mating-type-loci are mapped to two different chromosomes, with the *HML*α and *MAT* locus on chromosome B and the *HMR***a** on chromosome E. There is also an *HO* endonuclease site on the *α1* gene.

In the case of *C. glabrata*, probably the most phylogenetically close member of the *Nakaseomyces* to *S. cerevisiae*, three *MTL* loci and the *HO* gene have been previously reported in its genome, albeit the genome organisation is different than that of *S. cerevisiae* [75], with the *MAT* and *HML*-like cassette on chromosome B and the *HMR*-like cassette on chromosome E (Figure 7). In addition to isolates of both mating types being found, BG14 MAT**a** and CBS138 MATα are the most commonly utilised laboratory strains. Previous studies have set to examine the functionality of the genes involved in mating and meiosis in *C. glabrata*, [9,16,76] via expression profiling and functional complementation as there is population structure evidence of recombination and orthologues of the majority of the genes involved in fungal sexual reproduction, including many of those missing in other *Candida* species [76]. They found that the *MATα* gene α1 was expressed in all *MATα* strains and not in *MAT***a** strains as expected. However, the ***a****1* gene was expressed in both *MAT***a** and *MAT*α strains; therefore, it was concluded that the *HMR***a** locus on chromosome V was not silenced in *C. glabrata*. One could therefore hypothesize that the genome re-organisation seen in *C. glabrata* affects gene expression and this may play a role in the lack of a sexual cycle being observed. In addition to examining gene expression, the cell responses to pheromone have also been greatly studied. *C. glabrata* cells of both mating types express both ***a-*** and *α*- factor receptor genes, namely *STE2* and *STE3*, respectively. 

Similarly, in *N. delphensis*, a non-pathogenic haploid member of the *Nakaseomyces*, generally isolated from the environment, these elements, *HMR/HML*, and *HO* genome positions are also conserved [16]. Of the four remaining members of this group, two classified as pathogenic, *C. nivariensis* and *C. bracarensis* and the non-pathogen *C. castellii*, are considered asexual and haploid in nature. The fourth, *N. bacillisporus*, is considered to be diploid in nature. A series of important analysis has also been carried out on the sequence of the *HO* gene for all species in the *Nakaseomyces*. They were shown to be well conserved, with the most sequence divergence observed in *N. bacillisporus* [16,18,77]. The functionality of the *HO* gene is of importance as it encodes an endonuclease responsible for the initiation of mating-type switching, a process whereby a gene conversion changes *MAT***a** cells to *MAT*α or vice versa. As previously mentioned, the cell mating type is determined by information expressed from the *MAT* locus. *HO* expression and mating-type switching occurs exclusively in haploid mother cells at the end of G1 at the point that marks the commitment to a duplication event. This timing and transcription of *HO* is tightly regulated to ensure that switching only occurs in G1-arrested cells and not during mating. The majority of laboratory strains are ho minus and therefore are heterothallic; therefore, it is possible that by focusing our research on mating in the *Nakaseomyces* on laboratory-cultivated rather than wild-type strains, we are hindering the chances of observing natural mating in these species. One may therefore hypothesize that these yeasts, as they also contain the *HO* gene, are capable of switching mating types under laboratory conditions or have a cryptic sexual cycle. Similar to the chromosomal organisation of *S. cerevisiae*, the *Nakaseomyces* have retained the *MAT* loci and *HML/HMR* on the same chromosome. However, unlike in *S. cerevisiae*, one of the silent cassettes in *C. glabrata* is located on a different chromosome to the *MTL* and *HM*Lα. Could this different chromosomal organisation be a reason why no ‘natural’ mating in *C. glabrata* has been observed? However, rare recombination events have been identified [76]. 

## 10. Conclusions

While sexual cycles are ubiquitous throughout biology, and sexual identity is defined by *MAT*, which contains genes that regulate cell identity and sexual reproduction, mating can take different routes: Heterothallic, involving mating between partners with different mating type alleles, such as those well defined in *S. cerevisiae*; or homothallic, involving the reproduction of solo individual isolates (summary in Table 2). Homothallism can involve selfing or clonal reproduction. Sexual reproduction is seen as a diversity generator, being key to the generation of diversity in genotypes and phenotypes, leading to the re-assortment of alleles. Sexual reproduction can also contribute through the de novo diversity not currently observed in the population. This is an adaptive function in response to environmental changes in either nature or a host. 

One of the main questions that is continually investigated is why ‘natural’ mating is not observed in many fungal species and how have these species evolved to retain homologs of the genes essential for mating, sporulation, and meiosis? Is there a possible virulence role for the mating pathways beyond that of sex, for example, such as the formation of biofilms in *C. albicans*, often seen as a prelude to mating and the formation of drug resistant adherent biofilms. Have these genes been retained in the genomes of the *Nakaseomyces* as they are important for survival and evolving at a faster rate than non-sex genes? These are just some of the major questions that will for some time remain hotly debated and research-driven topics in the field. It is essential that we try to determine the functionality of these genes to determine whether they have evolved a novel function as they may have a role in pathogenicity.

## Figures and Tables

**Table 1:** List of genes involved in mating and meiosis.

*S. cerevisiae* Associated Genes	*Candida albicans* Associated Genes	*Candida glabrata* Associated Genes	*Aspergillus fumigatus* Associated Genes	Descriptions
IME1	-	CAGL0M09042g	-	Master regulator of meiosis that is active only during meiotic events; activates transcription of early meiotic genes through interaction with Ume6p, degraded by the 26S proteasome following phosphorylation by Ime2p
IME2	orf19.2395	CAGL0G04455g	Afu2g13140	Serine/threonine protein kinase involved in activation of meiosis; associates with Ime1p and mediates its stability, activates Ndt80p; IME2 expression is positively regulated by Ime1p
IME4	orf19.1476	CAGL0A03300g	Afu2g05600	mRNA N6-adenosine methyltransferase required for entry into meiosis; mediates N6-adenosine methylation of bulk mRNA during the induction of sporulation which includes the meiotic regulators IME1, IME2 and IME4 itself; repressed in haploids via production of antisense IME4 transcripts; transcribed in diploid cells where antisense transcription is repressed
KAR1	-	CAGL0J11418g	-	Protein involved in karyogamy and spindle pole body duplication; involved in karyogamy during mating; involved in spindle pole body duplication during mitosis
KAR3	orf19.564	CAGL0D04994g	Afu2g14280	Minus-end-directed microtubule motor; functions in mitosis and meiosis, localizes to the spindle pole body and localization is dependent on functional Cik1p, required for nuclear fusion during mating
KAR4	orf19.3736	CAGL0B00462g	-	Transcription factor required for response to pheromones; also required during meiosis; exists in two forms, a slower-migrating form more abundant during vegetative growth and a faster-migrating form induced by pheromone
MEK1	orf19.1874	CAGL0D02244g	Afu5g07950	Meiosis-specific serine/threonine protein kinase; functions in meiotic checkpoint, promotes recombination between homologous chromosomes by suppressing double strand break repair between sister chromatids; stabilizes Hop1-Thr318 phosphorylation to promote interhomolog recombination and checkpoint responses during meiosis
NDT80	orf19.2119	CAGL0L13090g	Afu2g09890	Meiosis-specific transcription factor; required for exit from pachytene and for full meiotic recombination; activates middle sporulation genes
RAD50	orf19.1648	CAGL0J07788g	Afu4g12680	Initiation of meiotic DSBs, telomere maintenance, and nonhomologous end joining
RAD51	orf19.3752	CAGL0I05544g	Afu1g10410	Strand exchange protein; forms a helical filament with DNA that searches for homology; involved in the recombinational repair of double-strand breaks in DNA during vegetative growth and meiosis
RIM11	orf19.791		Afu6g05120	
RME1	orf19.4438	CAGL0K04257g	-	Zinc finger protein involved in control of meiosis; prevents meiosis by repressing IME1 expression and promotes mitosis by activating CLN2 expression; directly repressed by a1-alpha2 regulator; mediates cell type control of sporulation
SET3	orf19.7221	CAGL0L03091g	Afu2g11210	Defining member of the SET3 histone deacetylase complex; which is a meiosis-specific repressor of sporulation genes; necessary for efficient transcription by RNAPII; one of two yeast proteins that contains both SET and PHD domains
SPO11	orf19.3589	CAGL0C02783g	Afu5g04070	Meiosis-specific protein that initiates meiotic recombination; initiates meiotic recombination by catalysing the formation of double-strand breaks in DNA via a transesterification reaction; required for homologous chromosome pairing and synaptonemal complex formation
SSP1	orf19.3173	CAGL0M13365g	Afu8g03930	Protein involved in the control of meiotic nuclear division; also involved in the coordination of meiosis with spore formation; transcription is induced midway through meiosis
STE2	orf19.696	CAGL0K12430g	Afu3g14330	Receptor for alpha-factor pheromone; seven transmembrane-domain GPCR that interacts with both pheromone and a heterotrimeric G protein to initiate the signalling response that leads to mating between haploid a and alpha cells
STE3	orf19.2492	CAGL0M08184g	Afu5g07880	Receptor for a factor pheromone; couples to MAP kinase cascade to mediate pheromone response; transcribed in alpha cells and required for mating by alpha cells
STE6	orf19.7440	CAGL0K00363g	Afu4g08800	Plasma membrane ATP-binding cassette (ABC) transporter; required for the export of a-factor, catalyses ATP hydrolysis coupled to a-factor transport; expressed only in MATa cells
STE7	orf19.469	CAGL0I03498g	Afu3g05900	Signal transducing MAP kinase; involved in pheromone response where it phosphorylates Fus3p; involved in the pseudohyphal/invasive growth pathway where it phosphorylates of Kss1p; phosphorylated by Ste11p
SUT1	orf19.4342	CAGLI04246g	Afu5g06210	Transcription factor of the Zn(II)2Cys6 family; positively regulates mating with SUT2 by repressing expression of genes which act as mating inhibitors
SUT2	-	CAGL0L09383g	-	Putative transcription factor of the Zn2Cys6 family; positively regulates mating along with SUT1 by repressing the expression of genes (PRR2, NCE102 and RHO5) which function as mating inhibitors
UME6	orf19.1822	CAGL0F05357g	Afu3g15290	Key transcriptional regulator of early meiotic genes; involved in chromatin remodelling and transcriptional repression via DNA looping; binds URS1 upstream regulatory sequence, couples metabolic responses to nutritional cues with initiation and progression of meiosis, forms complex with Ime1p

Genes with a role in mating and meiosis in *S. cerevisiae* are listed in addition to homologous genes from *C. albicans, C. glabrata*, and *A. fumigatus*. The absence of a homolog is marked by a dash (-).

**Table 2:** Summary of key characteristics of pathogenic fungi.

	*S. cerevisiae*	*C. albicans*	*C. glabrata*	*A. fumigatus*	*C. neoformans*
Lifestyle	Generally regarded as safe	Commensal and pathogenic	Commensal and pathogenic	Pathogenic	Pathogenic
Ploidy	Haploid in the majority of cases	Diploid with rare haploid strains observed	Haploid with aneuploidy observed in some clinical isolates	Haploid	Haploid with diploid blastospores able to undergo meiosis.
Mating genes	Present	Present	Present	Present	Present
Sexual reproduction	Homothallic	Asexual; Parasexual cycle observed	Not observed	Asexual; Rare sexual reproduction observed	Asexual; Rare sexual reproduction observed
Morphology	Yeast	Yeast, pseudohyphae and hyphae	Yeast	Conidiospores	Yeast, hyphae and basidiospores

## References

[1] de Pauw B.E. (2011). What are fungal infections?. Mediterr. J. Hematol. Infect. Dis..

[2] Perlin D.S., Rogers P.D., Howard S.J., Cowen L.E., Sanglard D. (2014). Mechanisms of Antifungal Drug Resistance. Cold Spring Harb. Perspect. Med..

[3] Brown G.D., Denning D.W., Gow N.A.R., Levitz S.M., Netea M.G., White T.C. (2012). Hidden Killers. Hum. Fungal Infect..

[4] Heitman J. (2012). Microbial Pathogens in the Fungal Kingdom. NIH Public Access..

[5] Nieuwenhuis B.P.S., James T.Y. (2016). The frequency of sex in fungi. Philos. Trans. R. Soc. B Biol. Sci..

[6] Butler G., Rasmussen M.D., Lin M.F., Santos M.A.S., Sakthikumar S., Munro C.A., Rheinbay E., Grabherr M., Forche A., Reedy J.L. (2009). Evolution of pathogenicity and sexual reproduction in eight *Candida* genomes. Nature.

[7] Ni M., Feretzaki M., Sun S., Wang X., Heitman J. (2011). Sex in Fungi. Annu Rev Genet..

[8] Butler G. (2010). Fungal sex and pathogenesis. Clin. Microbiol. Rev..

[9] Wong S., Fares M.A., Zimmermann W., Butler G., Wolfe K.H. (2003). Evidence from comparative genomics for a complete sexual cycle in the “asexual” pathogenic yeast Candida glabrata. Genome. Biol..

[10] Billiard S., López-Villavicencio M., Hood M.E., Giraud T. (2012). Sex, outcrossing and mating types: Unsolved questions in fungi and beyond. J. Evol. Biol..

[11] Billiard S., López-Villavicencio M., Devier B., Hood M.E., Fairhead C., Giraud T. (2011). Having sex, yes, but with whom? Inferences from fungi on the evolution of anisogamy and mating types. Biol. Rev..

[12] O’Gorman C.M., Fuller H.T., Dyer P.S. (2009). Discovery of a sexual cycle in the opportunistic fungal pathogen Aspergillus fumigatus. Nature.

[13] Sun S., Heitman J. (2011). Is sex necessary?. BMC Biol..

[14] Fu C., Sun S., Billmyre R.B., Roach K.C., Heitman J. (2014). Unisexual versus bisexual mating in Cryptococcus neoformans: Consequences and biological impacts. Fungal Genet. Biol..

[15] Halliday C.L., Carter D.A. (2003). Clonal reproduction and limited dispersal in an environmental population of Cryptococcus neoformans var. gattii isolates from Australia. J. Clin. Microbiol..

[16] Gabaldón T., Martin T., Marcet-Houben M., Durrens P., Bolotin-Fukuhara M., Lespinet O., Arnaise S., Boisnard S., Aguileta G., Atanasova R. (2013). Comparative genomics of emerging pathogens in the Candida glabrata clade. BMC Genom..

[17] Gabaldón T., Fairhead C. (2019). Genomes shed light on the secret life of *Candida* glarbrata, not so asexual, not so commensal. Current genetics..

[18] Enache-Angoulvant A., Guitard J., Grenouillet F., Martin T., Durrens P., Fairhead C., Hennequin C. (2011). Rapid discrimination between Candida glabrata, Candida nivariensis, and Candida bracarensis by use of a singleplex PCR. J. Clin. Microbiol..

[19] Torres E.M., Sokolsky T., Tucker C.M., Chan L.Y., Boselli M., Dunham M.J., Amon A., Yona A.H., Manor Y.S., Herbst R.H. (2013). Candida albicans and Candida glabrata clinical isolates exhibiting reduced echinocandin susceptibility. Antimicrob. Agents Chemother..

[20] Vershon A.K., Pierce M. (2000). Transcriptional regulation of meiosis in yeast. Curr opinion in cell boil..

[21] Kassir Y., Granot D., Simchen G. (1988). IME7, a Positive Regulator in S cerevisiae Gene of Meiosis. Cell.

[22] Strudwick N., Brown M., Parmar V.M., Schröder M. (2010). Ime1 and Ime2 are required for pseudohyphal growth of Saccharomyces cerevisiae on nonfermentable carbon sources. Mol. Cell. Biol..

[23] Arnone J.T., Robbins-Pianka A., Arace J.R., Kass-Gergi S., McAlear M. (2012). The adjacent positioning of co-regulated gene pairs is widely conserved across eukaryotes. BMC Genom..

[24] Magadum S., Banerjee U., Murugan P., Gangapur D., Ravikesavan R. (2013). Gene duplication as a major force in evolution. J. Genet..

[25] Forche A., Alby K., Schaefer D., Johnson A.D., Berman J., Bennett R.J. (2008). The parasexual cycle in Candida albicans provides an alternative pathway to meiosis for the formation of recombinant strains. PLoS Biol..

[26] Muller H., Hennequin C., Gallaud J., Dujon B., Fairhead C. (2008). The asexual yeast Candida glabrata maintains distinct a and α haploid mating types. Eukaryot. Cell.

[27] Irniger S. (2011). The Ime2 protein kinase family in fungi: More duties than just meiosis. Mol. Microbiol..

[28] Herskowitz I. (1988). Life cycle of the budding yeast Saccharomyces cerevisiae. Microbiol. Rev..

[29] Astell C.R., Ahlstrom-Jonasson L., Smith M., Tatchell K., Nasmyth K.A., Hall B.D. (1981). The sequence of the DNAs coding for the mating-type loci of saccharomyces cerevisiae. Cell.

[30] Hagen D.C., Bruhn L., Westby C.A., Sprague G.F. (1993). Transcription of alpha-specific genes in Saccharomyces cerevisiae: DNA sequence requirements for activity of the coregulator alpha 1. Mol. Cell. Biol..

[31] Zehr J., Heaney M., Sapiro V., Lo S. (2002). Letters To Nature. Nature.

[32] Galgoczy D.J., Cassidy-Stone A., Llinas M., O’Rourke S.M., Herskowitz I., DeRisi J.L., Johnson A.D. (2004). Genomic dissection of the cell-type-specification circuit in Saccharomyces cerevisiae. Proc. Natl. Acad. Sci. USA.

[33] Bowdish K.S., Yuan H.E., Mitchell A.P. (2015). Positive control of yeast meiotic genes by the negative regulator UME6. Mol. Cell. Biol..

[34] Smith H.E., Driscoll S.E., Sia R.A.L., Yuan H.E., Mitchell A.P. (1993). Genetic evidence for transcriptional activation by the yeast IME1 gene product. Genetics.

[35] Honigberg S.M., Purnapatre K. (2003). Signal pathway integration in the switch from the mitotic cell cycle to meiosis in yeast. J. Cell Sci..

[36] Yoshida M., Kawaguchi H., Sakata Y., Kominami K.I., Hirano M., Shima H., Akada R., Yamashita I. (1990). Initiation of meiosis and sporulation in Saccharomyces cerevisiae requires a novel protein kinase homologue. MGG Mol. Gen. Genet..

[37] Shah J.C., Clancy M.J. (1992). IME4, a gene that mediates MAT and nutritional control of meiosis in Saccharomyces cerevisiae. Mol. Cell. Biol..

[38] Guttmann-Raviv N., Martin S., Kassir Y. (2002). Ime2, a meiosis-specific kinase in yeast, is required for destabilization of its transcriptional activator, Ime1. Mol. Cell. Biol..

[39] Watanabe Y., Nurse P. (1999). Cohesin Rec8 is required for reductional chromosome segregation at meiosis. Nature.

[40] Richard G.F., Kerrest A., Lafontaine I., Dujon B. (2005). Comparative genomics of hemiascomycete yeasts: Genes involved in DNA replication, repair, and recombination. Mol. Biol. Evol..

[41] Tao L., Du H., Guan G., Dai Y., Nobile C.J., Liang W., Cao C., Zhang Q., Zhong J., Huang G. (2014). Discovery of a “White-Gray-Opaque” Tristable Phenotypic Switching System in Candida albicans: Roles of Non-genetic Diversity in Host Adaptation. PLoS Biol..

[42] Butler G., Kenny C., Fagan A., Kurischko C., Gaillardin C., Wolfe K.H. (2004). Evolution of the MAT locus and its Ho endonuclease in yeast species. Proc. Natl. Acad. Sci. USA.

[43] Paoletti M., Rydholm C., Schwier E.U., Anderson M.J., Szakacs G., Lutzoni F., Debeaupuis J.P., Latgé J.P., Denning D.W., Dyer P.S. (2005). Evidence for sexuality in the opportunistic fungal pathogen Aspergillus fumigatus. Curr. Biol..

[44] MacCallum D.M., Findon H., Kenny C.C., Butler G., Haynes K., Odds F.C. (2006). Different consequences of ACE2 and SWI5 gene disruptions for virulence of pathogenic and nonpathogenic yeasts. Infect. Immun..

[45] Calcagno A.M., Bignell E., Warn P., Jones M.D., Denning D.W., Mühlschlegel F.A., Rogers T.R., Haynes K. (2003). Candida glabrata STE12 is required for wild-type levels of virulence and nitrogen starvation induced filamentation. Mol. Microbiol..

[46] Calcagno A.M., Bignell E., Rogers T.R., Canedo M., Mühlschleger F.A., Haynes K. (2004). Candida glabrata Ste20 is involved in maintaining cell wall integrity and adaptation to hypertonic stress and is required for wild-type levels of virulence. Yeast.

[47] Heitman J. (2010). Evolution of eukaryotic microbial pathogens via covert sexual reproduction. Cell Host Microbe.

[48] Borneman A.R., Gianoulis T.A., Zhang Z.D., Yu H., Rozowsky J., Seringhaus M.R., Lu Y.W., Gerstein M., Snyder M. (2007). Divergence of transcription factor binding sites across related yeast species. Science.

[49] Losada L., Sugui J.A., Eckhaus M.A., Chang Y.C., Mounaud S., Figat A., Joardar V., Pakala S.B., Pakala S., Venepally P. (2015). Genetic Analysis Using an Isogenic Mating Pair of Aspergillus fumigatus Identifies Azole Resistance Genes and Lack of MAT Locus’s Role in Virulence. PLoS Pathog..

[50] Seider K., Gerwien F., Kasper L., Allert S., Brunke S., Jablonowski N., Schwarzmüller T., Barz D., Rupp S., Kuchler K. (2014). Immune evasion, stress resistance, and efficient nutrient acquisition are crucial for intracellular survival of Candida glabrata within macrophages. Eukaryot. Cell.

[51] Vale-Silva L., Ischer F., Leibundgut-Landmann S., Sanglard D. (2013). Gain-of-function mutations in PDR1, a regulator of antifungal drug resistance in candida glabrata, control adherence to host cells. Infect. Immun..

[52] Hörandl E. (2010). A combinational theory for maintenance of sex. Heredity (Edinb)..

[53] Robinson M.S., Reese T.S., De Camilli P., Rizo J., Gierasch L.M., Anderson R.G.W., Bonifacino J.S., Zuber J.F., Spiess M., Rapoport I. (1999). Identification of a Mating Type–Like Locus in the Asexual Pathogenic Yeast Candida albicans. Science.

[54] Fraser J.A., Heitman J. (2003). Fungal mating-type loci. Curr. Biol..

[55] (2019). Wilson; Wilken; van der Nest; Wingfield; Wingfield It’s All in the Genes: The Regulatory Pathways of Sexual Reproduction in Filamentous Ascomycetes. Genes (Basel)..

[56] Ene I.V., Bennett R.J., Anderson M.Z. (2019). Mechanisms of genome evolution in Candida albicans. Curr. Opin. Microbiol..

[57] Israeli E. (2013). The “obligate diploid” Candida albicans forms mating-competent haploids. Isr. Med. Assoc. J..

[58] Bennett R.J., Uhl M.A., Miller M.G., Johnson A.D. (2003). Identification and Characterization of a Candida albicans Mating Pheromone. Mol. Cell. Biol..

[59] Hull C.M., Raisner R.M., Johnson A.D. (2000). Evidence for mating of the “asexual” yeast Candida albicans in a mammalian host. Science.

[60] Glittenberg M.T., Kounatidis I., Christensen D., Kostov M., Kimber S., Roberts I., Ligoxygakis P. (2011). Pathogen and host factors are needed to provoke a systemic host response to gastrointestinal infection of Drosophila larvae by Candida albicans. Dis. Model. Mech..

[61] Miller M.G., Johnson A.D. (2002). White-opaque switching in Candida albicans is controlled by mating-type locus homeodomain proteins and allows efficient mating. Cell.

[62] (1987). “White-opaque transition”: A second high-frequency switching system in Candida albicans. J. Bacteriol..

[63] Huang G., Yi S., Sahni N., Daniels K.J., Srikantha T., Soll D.R. (2010). N-acetylglucosamine induces white to opaque switching, a mating prerequisite in Candida albicans. PLoS Pathog..

[64] Klar A.J.S. (2010). The yeast mating-type switching mechanism: A memoir. Genetics.

[65] Montelone B.A. (2003). Yeast Mating Type. ELS..

[66] Latgé J.P. (1999). Aspergillus fumigatus and Aspergillosis. Clin. Microbiol. Rev..

[67] Janbon G., Quintin J., Lanternier F., d’Enfert C. (2019). Studying fungal pathogens of humans and fungal infections: Fungal diversity and diversity of approaches. Genes Immun..

[68] Lengeler K.B., Fox D.S., Fraser J.A., Allen A., Forrester K., Dietrich F.S., Heitman J. (2002). Mating-type locus of Cryptococcus neoformans: A step in the evolution of sex chromosomes. Eukaryot. Cell.

[69] (2000). Mapping of the Cryptococcus neoformans MATα locus: Presence of mating type-specific mitogen-activated protein kinase cascade homologs. J. Bacteriol..

[70] Wickes B.L., Mayorga M.E., Edman U., Edman J.C. (1996). Dimorphism and haploid fruiting in Cryptococcus neoformans: Association with the alpha-mating type. Proc. Natl. Acad. Sci. USA.

[71] Laguna L., Disturbance P.B., Design M.J.E. (2005). Sexual reproduction between partners of the same mating type in Cryptococcus neoformans. Nature.

[72] Muller H., Thierry A., Coppée J.Y., Gouyette C., Hennequin C., Sismeiro O., Talla E., Dujon B., Fairhead C. (2009). Genomic polymorphism in the population of Candida glabrata: Gene copy-number variation and chromosomal translocations. Fungal Genet. Biol..

[73] Walsh D.W., Wolfe K.H., Butler G. (2002). Genomic differences between Candida glabrata and Saccharomyces cerevisiae around the MRPL28 and GCN3 loci. Yeast.

[74] Fitzpatrick D.A., O’Gaora P., Byrne K.P., Butler G. (2010). Analysis of gene evolution and metabolic pathways using the Candida Gene Order Browser. BMC Genom..

[75] Srikantha T., Lachke S.A., Soll D.R. (2003). Three mating type-like loci in Candida glabrata. Eukaryot. Cell.

[76] Dodgson A.R., Pujol C., Pfaller M.A., Denning D.W., Soll D.R. (2005). Evidence for recombination in Candida glabrata. Fungal Genet. Biol..

[77] Angoulvant A., Guitard J., Hennequin C. (2016). Old and new pathogenic Nakaseomyces species: Epidemiology, biology, identification, pathogenicity and antifungal resistance. FEMS Yeast Res..

